# Sex-specific cardiovascular risk factors in the UK Biobank

**DOI:** 10.3389/fphys.2024.1339866

**Published:** 2024-04-23

**Authors:** Skyler R. St. Pierre, Bartosz Kaczmarski, Mathias Peirlinck, Ellen Kuhl

**Affiliations:** ^1^ Department of Mechanical Engineering, Stanford University, Stanford, CA, United States; ^2^ Department of BioMechanical Engineering, Delft University of Technology, Delft, Netherlands

**Keywords:** cardiovascular, sex differences, risk factors, heart disease, UK Biobank

## Abstract

The lack of sex-specific cardiovascular disease criteria contributes to the underdiagnosis of women compared to that of men. For more than half a century, the Framingham Risk Score has been the gold standard to estimate an individual’s risk of developing cardiovascular disease based on the age, sex, cholesterol levels, blood pressure, diabetes status, and the smoking status. Now, machine learning can offer a much more nuanced insight into predicting the risk of cardiovascular diseases. The UK Biobank is a large database that includes traditional risk factors and tests related to the cardiovascular system: magnetic resonance imaging, pulse wave analysis, electrocardiograms, and carotid ultrasounds. Here, we leverage 20,542 datasets from the UK Biobank to build more accurate cardiovascular risk models than the Framingham Risk Score and quantify the underdiagnosis of women compared to that of men. Strikingly, for a first-degree atrioventricular block and dilated cardiomyopathy, two conditions with non-sex-specific diagnostic criteria, our study shows that women are under-diagnosed 2× and 1.4× more than men. Similarly, our results demonstrate the need for sex-specific criteria in essential primary hypertension and hypertrophic cardiomyopathy. Our feature importance analysis reveals that out of the top 10 features across three sexes and four disease categories, traditional Framingham factors made up between 40% and 50%; electrocardiogram, 30%–33%; pulse wave analysis, 13%–23%; and magnetic resonance imaging and carotid ultrasound, 0%–10%. Improving the Framingham Risk Score by leveraging big data and machine learning allows us to incorporate a wider range of biomedical data and prediction features, enhance personalization and accuracy, and continuously integrate new data and knowledge, with the ultimate goal to improve accurate prediction, early detection, and early intervention in cardiovascular disease management. Our analysis pipeline and trained classifiers are freely available at https://github.com/LivingMatterLab/CardiovascularDiseaseClassification.

## 1 Motivation

Historically, women have been excluded from the biomedical literature, and clinical and animal trials have been biased toward male-only or male-dominated populations (Garcia-Sifuentes and Maney, 2021). Including sex as a biological variable is increasingly recognized as being essential to decrease health inequities (Clayton and Collins, 2014; Cirillo et al., 2020). Sex is typically reported as a binary variable, but sex is inherently complex and relates to hormones, chromosomes, and physical characteristics, all of which follow distributions that overlap between the traditional *male* and *female* categories (Morgenroth and Ryan, 2021). In the UK Biobank, sex is reported as a binary variable and the language used in this study reflects that limitation (Sudlow et al., 2015).

### 1.1 Women are underdiagnosed and undertreated compared to men

Cardiovascular disease is underdiagnosed in women compared to that in men; the lack of sex-specific diagnostic criteria contributes to this issue (St. Pierre et al., 2022). With the currently used non-sex-specific criteria, the prevalence of dilated and hypertrophic cardiomyopathy is 3:1 and 3:2 for men-to-women, respectively, indicating that men are diagnosed more frequently than women for these cardiomyopathies (Olivotto et al., 2005; Cannatà et al., 2020). On average, women have a smaller wall thickness than men. For hypertrophic cardiomyopathy, the lack of sex-specific criteria implies that female hearts have to disproportionally increase more in thickness than male hearts to reach the diagnostic threshold of a wall thickness of 15 mm (van Driel et al., 2019). Strikingly, women are half as likely to be diagnosed during a routine examination for hypertrophic cardiomyopathy compared to men (Olivotto et al., 2005). Women are also diagnosed at an older age and with more symptoms than men for hypertrophic and dilated cardiomyopathies (Olivotto et al., 2005; Halliday et al., 2018; Cannatà et al., 2020).

The need for sex-specific diagnostic criteria is also visible in heart failure with the preserved left ventricle ejection fraction where the cut-off is an ejection fraction of ≥50% (Ponikowski et al., 2016). However, women have a higher baseline ejection fraction than men on average (Rutkowski et al., 2020). Studies have already shown that women benefit from therapies at a higher range of ejection fractions than men (McMurray et al., 2020; Solomon and McMurray, 2021). Clearly, there is an urgent need for sex-specific research to understand the different impacts of heart failure on men and women (Peirlinck et al., 2021b; Lala et al., 2022; Yang et al., 2023).

### 1.2 Risk prediction enables early detection

Clinically used risk prediction models for cardiovascular diseases typically include components of the Framingham Risk Score: age, sex, total cholesterol, high-density lipoprotein (HDL) cholesterol, systolic blood pressure, blood pressure treated through medicine, diabetes status, and smoking status (Wilson et al., 1998). The body mass index (BMI) is also common to include in risk models (Alaa et al., 2019). These risk models are easy to use; they only require a handful of easy-to-measure variables, and risk evaluation is a simple score based on discrete thresholds for each of these variables. Clinicians use these risk models to determine if an otherwise asymptomatic person would benefit from medical intervention (Alaa et al., 2019; Kremers et al., 2008; D’Agostino et al., 2008; Sjöström et al., 2004).

### 1.3 Machine learning models have historically outperformed deep learning for tabular data

Tabular data consist of features that can be input into a spreadsheet, including continuous variables, like the age and binary variables and the smoking status, which are coded with zero for negative and one for positive. The state-of-the-art approaches for supervised learning on tabular data are gradient-boosted tree ensembles (Borisov et al., 2022), which are conventional machine learning methods. The top gradient-boosted models based on benchmark performance for five independent datasets (Borisov et al., 2022) are XGBoost (eXtreme Gradient Boosting) (Chen and Guestrin, 2016), LightGBM (Ke et al., 2017), and CatBoost (Prokhorenkova et al., 2018). The strengths of tree ensemble methods include robustness against outliers and noisy data (Aceña et al., 2022) and the fast exact extraction of feature importance via methods such as SHapley Additive exPlanations (SHAP) (Lundberg and Lee, 2017). A weakness of decision trees is that they are unstable and tend to overfit the training data (Aceña et al., 2022).

Although gradient boosting frameworks have been shown to be the best-performing approaches for tabular data in the past decade (Shwartz-Ziv and Armon, 2022), deep learning architectures are becoming increasingly prevalent (Gorishniy et al., 2021), sometimes outperforming the state-of-the-art approaches (Somepalli et al., 2021; Borisov et al., 2022). In particular, deep learning frameworks, such as TabTransformer (Huang et al., 2020), DeepFM (Guo et al., 2017), TabNet (Arik and Pfister, 2020), and SAINT (Self-Attention and Intersample Attention Transformer) (Somepalli et al., 2021), showed significant promise for effective tabular data modeling, with the SAINT model outperforming the gradient-boosted frameworks on some learning tasks using the power of representation learning (Somepalli et al., 2021; Borisov et al., 2022). Nonetheless, a well-known downside of deep learning models, as compared to tree-based frameworks, is that they are generally much slower to train (Grinsztajn et al., 2022).

### 1.4 Machine learning can discover the most predictive features for risk models

Machine learning models can effectively utilize a large number of input features, which allows them to discover new, more accurate risk models to better identify people at risk (Madani et al., 2018; Alaa et al., 2019; Alber et al., 2019). XGBoost has been applied extensively for cardiovascular disease diagnoses (Rajliwall et al., 2018; Alaa et al., 2019; Athanasiou et al., 2020; Rajadevi et al., 2021; Papadopoulou et al., 2022). Prior statistical or deep learning models of cardiovascular diseases focused on the lifestyle factors (Sjöström et al., 2004; Alaa et al., 2019; Papadopoulou et al., 2022; Sharma et al., 2022), medical history (Alaa et al., 2019; Papadopoulou et al., 2022), sociodemographics (Alaa et al., 2019; Papadopoulou et al., 2022), dietary and nutritional information (Alaa et al., 2019; Papadopoulou et al., 2022), genetics (Papadopoulou et al., 2022; Sharma et al., 2022), and/or one of the four clinical tests: pulse wave analysis (Davies and Struthers, 2005; Said et al., 2018), electrocardiograms (Attia et al., 2019; Ramírez et al., 2021; Papadopoulou et al., 2022), carotid ultrasounds (Zhao et al., 2016; Sharma et al., 2022), or magnetic resonance imaging (Chung et al., 2006; Gilbert et al., 2019; Bai et al., 2020), but not all four.

### 1.5 Objectives of this study

First, we investigate whether women are underdiagnosed for cardiovascular diseases in the UK Biobank cohort. Then, we compare the performance of three models, a multilayer perceptron deep learning baseline, XGBoost, and the novel deep learning framework, SAINT, on their ability to predict whether a person can be diagnosed with a cardiovascular disease. Lastly, we identify the top sex- and disease-specific risk factors from four cardiovascular-related tests, pulse wave analysis, electrocardiograms, magnetic resonance imaging, and carotid ultrasounds, against the traditional Framingham Risk Score.

## 2 Materials and methods

### 2.1 Dataset and features

The UK Biobank comprises data from half a million individuals from the UK who were over the age of 40 (Sudlow et al., 2015). From these, we selected individuals who underwent ECG testing, magnetic resonance imaging, carotid ultrasounds, and pulse wave analysis, resulting in a population of 20,542 individuals. We also pulled features associated with the Framingham Risk Score, sex, age, total cholesterol, HDL cholesterol, smoking status, diabetes status, end-systolic blood pressure, the body mass index of all participants, and their medical diagnoses. Table 1 shows the demographic data on this population. We did not include treatment for blood pressure as a feature in our models as this directly reflects one of the diagnostic outcomes, hypertension, that we are trying to predict. All 57 features are shown in Table 2.

**TABLE 1:** Demographic data. Sex differences for the population of 20,542 individuals used in this study categorized by the Framingham Risk Score features.

	Age (years)	BMI (−)	Total cholesterol (mmol/L)	HDL cholesterol (mmol/L)	Smoker (−)	Diabetic (−)	Systolic blood pressure (mmHg)
Female, 10,585	62.82 ± 7.41	25.88 ± 4.51	5.83 ± 1.07	1.64 ± 0.37	3,586	340	113.74 ± 18.98
Male, 9,957	63.95 ± 7.61	26.72 ± 3.64	5.60 ± 1.07	1.31 ± 0.30	3,991	662	114.05 ± 16.38

The mean and standard deviation are reported for age, body mass index (BMI), total cholesterol, HDL cholesterol, and end-systolic blood pressure. Fisher’s exact test is used for the categorical features, while the Wilcoxon rank-sum test is used for all other features to determine if the distributions of the male and female populations are significantly different; ^†^ indicates *p* <0.05.

**TABLE 2:** Features for inclusion in the risk prediction analysis. The traditional Framingham Risk Score and body mass index are commonly used factors in clinical risk prediction for cardiovascular diseases. Features from magnetic resonance imaging, carotid ultrasounds, electrocardiogram recordings, and pulse wave analysis are also extracted from the UK Biobank.

Method	Features
Framingham Risk Score + body mass index (8)	Sex, age, high-density lipoprotein cholesterol, total cholesterol, end systolic blood pressure, smoking status, diabetes status, body mass index
Magnetic resonance imaging (7)	Average heart rate, cardiac index, cardiac output, left ventricle ejection fraction, left ventricle end-diastolic volume, left ventricle end-systolic volume, left ventricle stroke volume
Carotid ultrasound (12)	Max/mean/min carotid intima-media thickness 120/150/210/240
Electrocardiogram (12)	Ventricular rate, P duration, PP interval, PQ interval, QRS number, QRS duration, QT interval, QTC interval, RR interval, P axis, R axis, T axis
Pulse wave analysis (19)	Position of pulse wave notch, position of pulse wave peak, position of shoulder on pulse waveform, pulse rate, pulse wave arterial stiffness index, pulse wave peak to peak time, pulse wave reflection index, augmentation index, central augmentation pressure, central pulse pressure, central systolic blood pressure, diastolic blood pressure, end-systolic pressure index, mean arterial pressure index, number of beats in waveform average, peripheral pulse pressure, stroke volume, systolic brachial blood pressure

We created two feature groups: 1) eight features, including Framingham Risk Score features and the body mass index only, and 2) all 57 features. We labeled each person to be in the positive class if they were diagnosed with a given cardiovascular disease (Said et al., 2018). We split the datasets based on four disease categories, as shown in Table 3, namely, any disease, hypertension (ICD-10 codes I10–I15), ischemic (I20–I25), and conduction disorders (I44–I49) (World Health Organization, 2004), such that the detection of each disease can pose as a binary classification. We chose these disease categories because they had the largest number of participants who both had these diagnoses and data from all four imaging studies, ECG, heart MRI, pulse wave analysis, and carotid ultrasounds. To train the sex-specific classifiers, we further designated three input groups, i.e., both sexes, female only, and male only.

**TABLE 3:** Disease classification criteria. Clinical diagnostic ICD-10 codes for different subsets of cardiovascular disease (Said et al., 2018).

Disease	ICD10 codes
Any cardiovascular disease	I00-I78.9, G95.1, H334.1-2, O10.0-9, S06.60-61, Z95.1, Z95.5
Hypertensive diseases	I10-I15.9
Ischemic diseases	I20-I25.9
Conduction disorders	I44-I49.9

**FIGURE 1:**
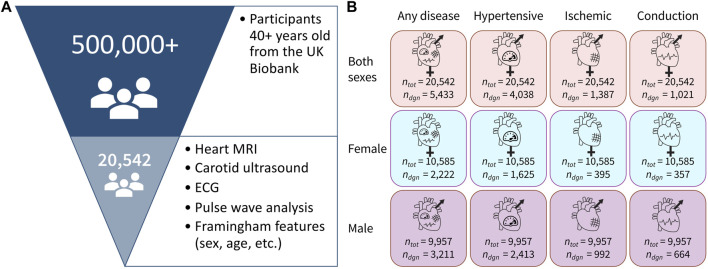
Dataset overview. **(A)** Out of 500,000+ participants in the UK Biobank study, we selected a group of 20,542 participants who underwent magnetic resonance imaging, carotid ultrasounds, ECG, and pulse wave analysis. We also selected participants with available data for all of the Framingham Risk Score features. **(B)** Data are separated in 12 variants with three sex groups and four cardiovascular disease categories, where *n*_*tot*_ is the total number of people in the dataset and *n*_diag_ is the number of people in that dataset who have been diagnosed with the corresponding condition.

Using the three input groups (both sexes, female only, and male only) together with the four binary label sets (any disease, hypertensive disease, ischemic heart disease, and conduction disorders), we constructed 12 dataset variants to train the binary classifiers, as shown in Figure 1. We generated each of the datasets by the direct slicing of the randomly pre-shuffled data frame. Since the datasets are relatively small, we applied a 70–15–15 split to create the training, validation, and test sets for each of the variants. The positive class in all 12 dataset variants is significantly underrepresented relative to the negative class, so we applied oversampling to approximately equalize the number of negative and positive samples in the training sets of the corresponding 12 datasets.

### 2.2 Models

Using the cardiovascular and Framingham Risk Score features, we implemented three distinct model types: 1) a multilayer perceptron (MLP); 2) an XGBoost ensemble model, which is a state-of-the art approach for tabular data learning (Chen and Guestrin, 2016); and 3) the SAINT model (Somepalli et al., 2021). We used the MLP as a baseline for deep learning performance and the XGBoost as a baseline for a state-of-the-art performance. For each model type, we trained and evaluated 12 individual classifiers, according to our 12 dataset variants. For evaluation purposes, we consider both untuned and tuned XGBoost ensemble models and introduce an additional set of tuned XGBoost ensembles trained only on the Framingham Risk Score features. The 12 cardiovascular disease datasets, three model types, and two additional XGBoost variants result in a total of 60 individually trainable classifiers.

#### 2.2.1 MLP: a deep learning model for a baseline comparison

We implemented and evaluated an MLP network under TensorFlow (Abadi et al., 2016) for each of the 12 dataset variants. All 12 MLP classifiers were trained using the binary cross-entropy function with *L*_2_ regularization of the cost. To accelerate and stabilize training, the features are first passed through a standardization layer, and each hidden layer is followed by a batch normalization layer. Each layer uses ReLU non-linearity, and the output uses a sigmoid activation function. The tunable hyperparameters of the MLP are the number of hidden layers, the number of units in each hidden layer, the *L*_2_-regularization parameter, and the parameters of the training procedure. Appendix A provides additional details about the MLP architecture and hyperparameter tuning.

#### 2.2.2 XGBoost: a state-of-the-art analysis of tabular data

We used XGBoost (Chen and Guestrin, 2016) as a benchmarking baseline for the novel SAINT model. XGBoost trains an ensemble of decision tree models using an efficient second-order gradient boosting framework (Chen and Guestrin, 2016). We trained an individual XGBoost ensemble for each of the 12 dataset variants using the binary cross-entropy loss and training-test splits consistent with those used for MLP models. Since XGBoost is a state-of-the-art approach for tabular data learning, we include both the tuned and untuned XGBoost ensembles for each dataset. The untuned XGBoost models represent the out-of-the-box performance of the current state-of-the-art method, while the performance of the tuned XGBoost models represents the best-case learning result in each dataset. Appendix A provides details of hyperparameter tuning for XGBoost models.

#### 2.2.3 SAINT: a novel approach for tabular data learning

The SAINT is a novel approach for tabular data modeling that employs self-attention, intersample attention, an enhanced embedding framework, and a contrastive pre-training phase (Somepalli et al., 2021). Transformers are a recent machine learning development that utilizes a multi-head attention mechanism, allowing the parallelized computation of the contextual representations of the input data. This new architecture is employed in cutting-edge generative machine learning applications, such as ChatGPT-4 (OpenAI, 2023). They have been shown to significantly outperform the previous state-of-the-art machine learning architecture for language modeling and machine translation tasks (Vaswani et al., 2017). Rather than using transformers for language processing purposes, SAINT adapts this architecture and the concept of self-attention to perform efficient learning on tabular data, such as the clinical UK Biobank data analyzed in our study. The SAINT architecture consists of multiple stages, each of which includes a self-attention block and an intersample attention block. The self-attention block applies attention on the features of a given sample, while intersample attention applies row-wise attention across different samples for a given feature. As a result, the final SAINT stage outputs a contextual representation of input embedding. Appendix B provides further information about the definitions, structure, and implementation of the SAINT framework (Somepalli et al., 2021). Although SAINT has been shown to outperform the state-of-the-art methods on some datasets (Borisov et al., 2022), it has not yet been used for cardiovascular data learning. Here, we applied SAINT to investigate its performance in cardiovascular disease classification tasks, in addition to the more established MLP and XGBoost methods.

### 2.3 Model evaluation

#### 2.3.1 ROC: receiver operating characteristic curve

The receiver operating characteristic (ROC) curve is used to evaluate the performance of a diagnostic test where the predictors of the outcome are not binary, so there are many possible cut-points to classify a person with a positive or negative diagnosis (Mandrekar, 2010). The ROC curve is a plot of sensitivity (true positive rate) vs. 1−specificity (false positive rate). Sensitivity is the probability that an individual who is truly positive gets a positive test result, while specificity is the probability that an individual who is truly negative gets a negative test result (Parikh et al., 2008). The diagonal line indicates that whether or not a person is diagnosed is totally random.

#### 2.3.2 AUC: area under the curve

The area under the curve (AUC) is a summary metric for the ROC curve that reports the overall accuracy of the test (Mandrekar, 2010). The AUC ranges from 0, completely inaccurate, to 1, completely accurate, with an AUC of 0.5, which means that the test result is random. We used the ROC curve and AUC metric to compare how accurately our different models can predict cardiovascular disease as the ROC curve does not depend on the scale of the test results and provides a helpful visual comparison (Mandrekar, 2010).

#### 2.3.3 Feature importance rankings

SHAP is a unified framework designed to interpret model predictions by giving a value for the importance of each feature to a specific prediction (Lundberg and Lee, 2017). A positive SHAP value indicates that a feature has a positive impact on the prediction of the positive class, which, in our case, is a diagnosis of a cardiovascular disease, while a negative SHAP value indicates the opposite. The magnitude indicates the strength of the effect. We can easily integrate the SHAP pipeline with XGBoost using the TreeExplainer class.

### 2.4 Calculation of an underdiagnosis

An underdiagnosis is calculated as the ratio of the number of people who, at a single time point, met the criteria for a given disease diagnosis but were never diagnosed for that disease across the entire timespan of the medical record divided by the number of people who had been diagnosed for the same disease at any point in time across the entire span of their medical records. The UK Biobank ICD-10 medical records start between 1981 and 1988, depending on the country in the UK, and last until 2022. Only 4% of the UK Biobank population has ICD-9 records, so we have not included these. The blood pressure was measured between 2006 and 2010, and data from the first time point of imaging visits, ECG, MRI, carotid ultrasound, and pulse wave analysis were collected after 2014. The *numerator* represents the *minimum possible value*; it implies that at a single time point between 1981 and 2022, people met the disease criteria but never got a diagnosis. It is a minimum because there are likely people from the healthy category who, at any other time point, would have met the criteria but were not measured at that precise time and were also never diagnosed. The *denominator* represents the *maximum possible value*; it reflects whether people have ever been diagnosed across the entire timeline from 1981 to 2022. So, this metric of underdiagnosis is in itself an *under*estimation.

## 3 Results

### 3.1 Women are underdiagnosed relative to men

We first investigate whether women are underdiagnosed relative to men for cardiovascular diseases in the UK Biobank cohort. We chose diseases where the diagnosis is a simple, non-sex-specific cut-off.

**FIGURE 2:**
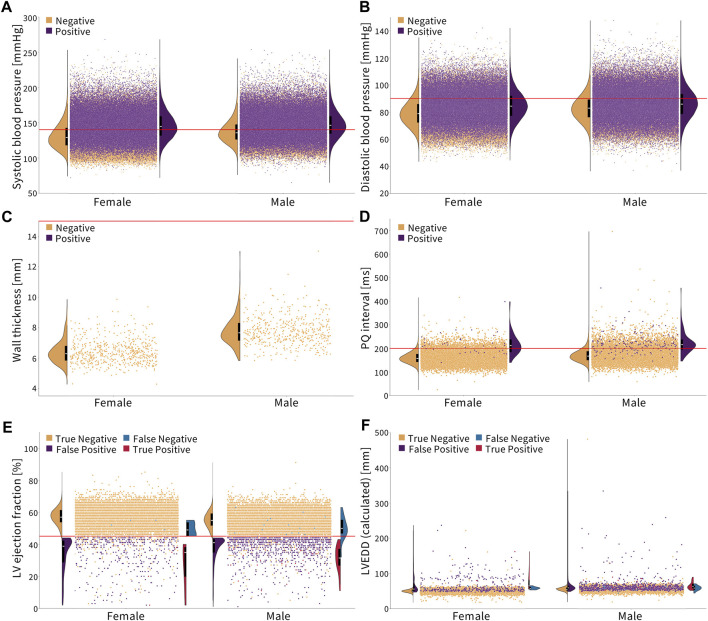
Diagnosing cardiovascular disease via simple, non-sex-specific cut-offs. The red line indicates the diagnostic cut-off. The truncated violin plots show the distribution of men and women for each color-coded population, with the box plot inside showing the mean in white and the 25th and 75th percentiles. **(A, B)** Essential primary hypertension is diagnosed with a systolic blood pressure greater than or equal to 140 mmHg and/or diastolic blood pressure greater than or equal to 90 mmHg (Williams et al., 2018). Women who are not diagnosed with hypertension, on average, have a lower systolic and diastolic blood pressure compared to men. **(C)** Hypertrophic cardiomyopathy is diagnosed with a wall thickness greater than 15 mm (Elliott et al., 2014). None of the individuals in this cohort met the condition. Healthy women have a notably lower wall thickness on average than men. **(D)** First-degree AV block is diagnosed with a PQ interval greater than 200 ms (Holmqvist and Daubert, 2013). Healthy women have a lower PQ interval on average than men. **(E, F)** Dilated cardiomyopathy is diagnosed by a left ventricle ejection fraction less than 45% and a left ventricle end-diastolic diameter greater than 112% of the diameter predicted based on the body surface area and age (Arora et al., 2010; Orphanou et al., 2022). Women have a slightly higher ejection fraction and lower left ventricle end-diastolic diameter on average than men, which is represented in orange.

Figure 2 shows the cut-off criteria in red. Plots (a–d) show individuals who have not been diagnosed with the disease in orange and those who have in purple. Plots (e,f) are divided into four categories. The truncated violin plots show the distribution of each sex for each category with the box plots showing the mean in white and the 25th–75th percentiles. Each dot represents a single person in the Biobank dataset. There may be comorbidities or alternate medical diagnoses that result in similar presentations, so the magnitude of an underdiagnosis, in the following examples, should be understood as a first approximation.

*Essential primary hypertension* is diagnosed by a systolic blood pressure of ≥140 mmHg and/or diastolic blood pressure of ≥90 mmHg (Williams et al., 2018). The corresponding ICD-10 code is I10. Figures 2A,B show that women have, on average, a lower systolic and diastolic blood pressure than men. In the Biobank cohort, 35.2% of men are diagnosed, while 52.3% of men meet the cut-off criteria. For women, 26.6% are diagnosed, while 40.2% meet the cut-off criteria. This means that women and men are underdiagnosed for essential primary hypertension at the same rate, 1.5×, when non-sex-specific criteria are used.

*Hypertrophic cardiomyopathy* is diagnosed with a wall thickness of > 15 mm (Elliott et al., 2014); the ICD-10 codes are I42.1 and I42.2. Figure 2C shows that none of the approximately 900 people with cardiac magnetic resonance images met the criteria for hypertrophic cardiomyopathy or were diagnosed. Women, on average, have a distinctly smaller wall thickness than men.

The *first-degree AV block* is diagnosed with a PQ interval of > 200 ms (Holmqvist and Daubert, 2013); the ICD-10 code is I44.0. As shown in Figure 2D, women have a smaller PQ interval than men on average. The first-degree AV block is generally asymptomatic but is no longer considered entirely benign, with nearly double the risk of developing atrial fibrillation and triple the risk of needing a pacemaker (Holmqvist and Daubert, 2013). As such, the current recommendation is to monitor patients regularly to see if the conduction delay continues to widen or if they are developing atrial fibrillation (Oldroyd et al., 2022). In the UK Biobank, 0.81% of men are diagnosed and 12.6% of men meet the cut-off. For women, 0.18% of them are diagnosed, while 5.4% meet the cut-off. So, women are underdiagnosed 30×, while men are underdiagnosed 15.6×, meaning that women are nearly 2× more underdiagnosed relative to men for a first-degree AV block with the given non-sex-specific criteria.

*Dilated cardiomyopathy* is diagnosed by a left ventricle ejection fraction of < 45% and a left ventricle end-diastolic diameter of > 112% of the predicted diameter based on the age and sex (Orphanou et al., 2022). Left ventricle fractional shortening less than 25% can be used in place of the ejection fraction criteria, but these data were not available in the Biobank. Because the left ventricle end-diastolic volume is reported, we used the Teichholz formula (Arora et al., 2010), $\text{LVEDV}=7{\left({\text{LVEDD}}_{\text{cal}}\right)}^{3}/\left(2.4+{\text{LVEDD}}_{\text{cal}}\right)$ , to calculate the end-diastolic diameter from the volume and the formula, LVEDD_pre_ = 45.3(BSA)^0.3^–0.03 (age) − 7.2, to predict the end-diastolic diameter from the BSA and age. If LVEDD_cal_/LVEDD_pre_ >1.12, the individual would meet the criteria and either be assigned a red or purple dot, as shown in Figure 2E,F, depending on whether they had also been diagnosed with dilated cardiomyopathy or not, respectively. If they did not meet this criterion, they were assigned an orange or blue dot, where blue indicates that they had been diagnosed and orange indicating that they had not been. In the Biobank cohort, 55 people were diagnosed with dilated cardiomyopathy, but only 35 met the cut-off using these calculations, with nearly all of these discrepancies for not meeting the ejection fraction criteria, as shown by the blue dot. Women, on average, have a slightly lower end-diastolic diameter and higher ejection fraction than men. Out of the men in the cohort, 0.23% of them were diagnosed, while 5.71% met the cut-off criterion. For women, 0.06% of them were diagnosed, while 2.02% met the cut-off criterion. As such, women are underdiagnosed 33.7×, men are underdiagnosed 24.8×, and women are 1.4× more underdiagnosed than men when non-sex-specific criteria are used.

### 3.2 The SAINT model performs the best in predicting risks for cardiovascular diseases

**FIGURE 3:**
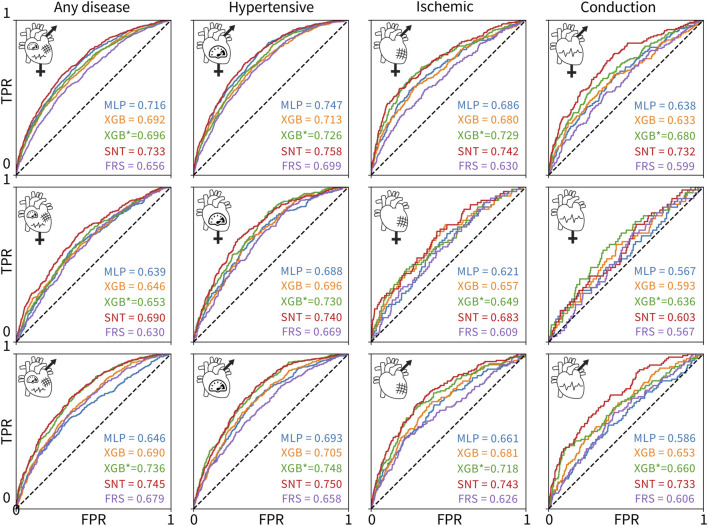
ROC curves and AUC scores for the 60 classifiers evaluated on 12 test sets. The rows correspond to (1) both sexes, (2) female-only, and (3) male-only datasets. The columns correspond to the (1) any, (2) hypertensive, (3) ischemic, and (4) conduction diseases. The colors of the curves indicate the different model types: MLP deep learning baseline (blue), untuned XGBoost (orange), tuned XGBoost baseline for the state-of-the-art model (green), SAINT (red), and XGBoost trained and tuned on Framingham Risk Score features only (purple). The true positive rate is plotted versus the false positive rate.

Figure 3 shows the ROC and AUC values for the five model types, MLP, untuned XGBoost, tuned XGBoost, SAINT, and XGBoost, with only Framingham Risk Score features across the 12 sex and disease categories. A large AUC score is designed to minimize false negatives (predicted healthy but actually diseased) and maximize true positives (predicted diseased and actually diseased). Table 4 summarizes the performance metrics for all classifiers, where we report the test set accuracy, precision, and recall in addition to the AUC score. In terms of the AUC metric, the SAINT model performed best on all datasets, except the female-only conduction disorder datasets, where only the corresponding tuned XGBoost model performed better. For accuracy and precision, XGBoost (tuned) models were the best performing in 11/12 cases and 8/12 cases, respectively. The SAINT had the best-performing recall in 9/12 cases.

**TABLE 4:** Comparison of 60 classifiers. For each test set, we report the accuracy (Acc), precision (Prec), recall (Rec), and area under the curve (AUC) scores for each of the five evaluated model types: MLP, untuned XGBoost, tuned XGBoost, SAINT, and XGBoost trained and tuned with Framingham Risk Score features only.

		MLP	XGBoost (untuned)	XGBoost (tuned)	SAINT	XGBoost (Fram. only)
Both sexes, any disease	Acc:	0.652	0.704	**0.732**	0.682	0.707
	Prec:	0.426	0.473	**0.551**	0.455	0.469
	Rec:	**0.667**	0.450	0.268	0.651	0.291
	AUC:	0.716	0.692	0.696	**0.733**	0.656
Both sexes, hypertension	Acc:	0.663	0.747	**0.786**	0.633	0.767
	Prec:	0.354	0.410	**0.498**	0.343	0.417
	Rec:	0.695	0.420	0.212	**0.780**	0.226
	AUC:	0.747	0.713	0.726	**0.758**	0.699
Both sexes, ischemic	Acc:	0.765	0.906	**0.931**	0.677	0.919
	Prec:	0.147	0.243	**0.542**	0.137	0.207
	Rec:	0.491	0.162	0.060	**0.681**	0.056
	AUC:	0.686	0.680	0.729	**0.742**	0.630
Both sexes, conduction	Acc:	0.765	0.934	**0.952**	0.739	0.946
	Prec:	0.085	0.184	**0.500**	0.101	0.050
	Rec:	0.396	0.107	0.027	**0.557**	0.007
	AUC:	0.638	0.633	0.680	**0.732**	0.599
Female, any disease	Acc:	0.634	0.741	**0.761**	0.692	0.734
	Prec:	0.336	0.417	**0.462**	0.378	0.341
	Rec:	**0.591**	0.287	0.182	0.504	0.157
	AUC:	0.639	0.646	0.653	**0.690**	0.630
Female, hypertension	Acc:	0.716	0.797	**0.837**	0.770	0.799
	Prec:	0.283	0.335	**0.527**	0.354	0.298
	Rec:	0.469	0.237	0.111	**0.477**	0.160
	AUC:	0.688	0.696	0.730	**0.740**	0.669
Female, ischemic	Acc:	0.857	**0.955**	0.951	0.700	0.948
	Prec:	0.089	**0.333**	0.167	0.075	0.000
	Rec:	0.243	0.029	0.029	**0.514**	0.000
	AUC:	0.621	0.657	0.649	**0.683**	0.609
Female, conduction	Acc:	0.880	0.965	**0.965**	0.945	0.964
	Prec:	0.070	**0.250**	0.000	0.075	0.000
	Rec:	**0.204**	0.019	0.000	0.056	0.000
	AUC:	0.567	0.593	**0.636**	0.603	0.567
Male, any disease	Acc:	0.603	0.675	**0.709**	0.669	0.687
	Prec:	0.434	0.518	**0.641**	0.505	0.552
	Rec:	0.609	0.426	0.296	**0.668**	0.348
	AUC:	0.646	0.690	0.736	**0.745**	0.679
Male, hypertension	Acc:	0.662	0.718	**0.755**	0.690	0.722
	Prec:	0.406	0.459	**0.603**	0.444	0.461
	Rec:	0.589	0.356	0.222	**0.677**	0.285
	AUC:	0.693	0.705	0.748	**0.750**	0.658
Male, ischemic	Acc:	0.723	0.878	**0.893**	0.751	0.890
	Prec:	0.167	0.262	0.257	0.209	**0.267**
	Rec:	0.476	0.154	0.063	**0.573**	0.084
	AUC:	0.661	0.681	0.718	**0.743**	0.626
Male, conduction	Acc:	0.807	0.925	**0.932**	0.612	0.922
	Prec:	0.100	**0.281**	0.250	0.114	0.087
	Rec:	0.245	0.092	0.020	**0.724**	0.020
	AUC:	0.586	0.653	0.660	**0.733**	0.606

Although all 60 models were measurably better than a random classifier, none of the models demonstrated a high AUC score. Since we did not observe training set overfitting, this might be indicative of a high Bayes error rate and low feature-output correlations in the datasets. The ischemic disease and conduction disorder models performed rather poorly, most likely caused by the small training set sizes and the increasingly significant class imbalance in their test sets.

### 3.3 Additional features improve cardiovascular disease prediction

Figure 3 suggests that including features from ECG, magnetic resonance imaging, pulse wave analysis, and carotid ultrasound, along with Framingham Risk Score features significantly increased the AUC score of the corresponding 48 models, as compared to the AUC scores for the Framingham-only XGBoost models. The XGBoost classifiers trained and tuned on the Framingham-only features were always the lowest or second-lowest performing models for a given dataset.

### 3.4 Predicting the risk of cardiovascular disease for women is less accurate than that for men

**FIGURE 4:**
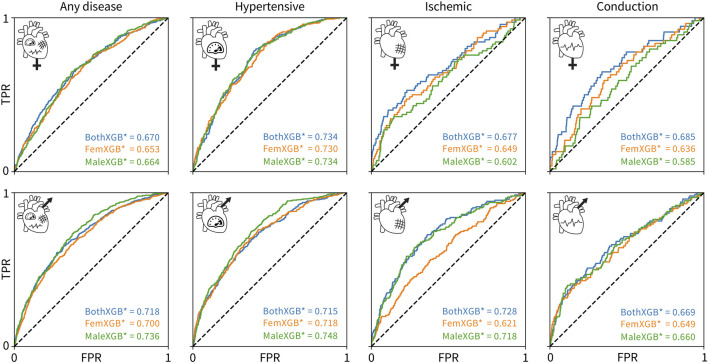
Cross-evaluation results using tuned XGBoost classifiers. The classifiers trained on both sexes are colored blue, the classifiers trained on only female data are colored orange, and the classifiers trained on only male data are colored green. The rows show the ROC and AUC for a given trained classifier in predicting a given disease for only-female data, top, or only-male data, bottom. The columns correspond to any cardiovascular disease, hypertensive diseases, ischemic diseases, and conduction diseases, from left to right. The true positive rate is plotted versus the false positive rate.

Figure 4 shows the performance of the 12 individually trained XGBoost classifiers on individual sexes. First, the classifiers trained on both sexes perform the best for all female-only datasets, top row. The best AUC values for the female-only data are lower than the best AUC values for the male-only data for all disease categories, except conduction disorders. Second, the male-only classifiers perform the best for the male-only datasets for any disease and hypertension categories, while the both-sex classifiers perform the best for ischemic and conduction diseases. Third, the performance of all classifiers is fairly similar for most sex- and disease-specific categories, except for three cases: 1) the female-only classifier is significantly worse at predicting male cases of ischemic diseases, 2) the male-only classifier is worse at predicting female cases of ischemic diseases, and 3) at predicting conduction diseases as compared to the female-only and both-sex classifiers.

### 3.5 A subset of the Framingham Risk Score and ECG features is the most predictive for cardiovascular disease

**FIGURE 5:**
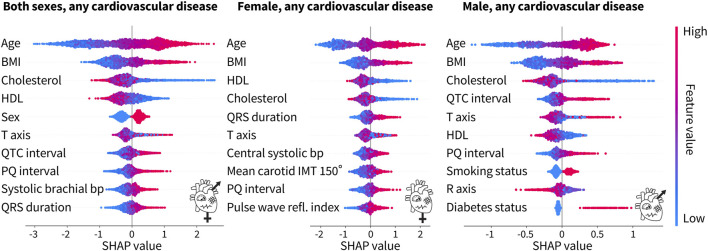
Top 10 features from the tuned XGBoost classifiers trained on both sexes, female only, and male only for any cardiovascular disease. For both sexes, the top four features for the prediction of cardiovascular disease are traditional risk factors, while ECG features and a blood pressure feature from pulse wave analysis make up the rest of the top 10. For the female-only dataset, in addition to the top four traditional risk factors, there is a mix of ECG, pulse wave, and carotid ultrasound features. For the male-only dataset, six of the features are traditional risk factors while the rest are ECG features. Each dot corresponds to a person in the SHAP analysis dataset. A positive SHAP value indicates the contribution to a diagnosis of cardiovascular disease. Bright red corresponds to a high feature value, e.g., old age, while bright blue corresponds to a low feature value, e.g., young age. The binary categories of sex, smoking status, and diabetes status are red for male, smoker, and diabetic, respectively, while blue represents the opposite.

Figure 5 shows the 10 most predictive features for any type of cardiovascular disease for both sexes combined, women only, and men only. A more positive SHAP value indicates a larger contribution to the positive class, diagnosed with a cardiovascular disease, while a negative SHAP value indicates the opposite. Each dot represents an individual person in the dataset, while the red color means that a person had a high value of that feature, e.g., older, while blue means a lower value, e.g., younger. For the binary categories of sex, smoking status, and diabetes status, red represents a male subject, a person who smokes, and a person with diabetes, respectively. Traditional risk factors refer to the Framingham Risk Score features plus body mass index, as shown in Table 2.

For both sexes combined, six of the top 10 features, namely, age, body mass index, cholesterol, HDL cholesterol, sex, and blood pressure, are the factors that are traditionally associated with an increased cardiovascular risk. The other four features are ECG features. For the female-only dataset, five of the top 10 features are traditional risk factors but the rest of the features include a mix of ECG, pulse wave analysis, and carotid ultrasound features. For the male-only dataset, six of the traditional risk factors make up the top 10 features. The other four features are ECG features. Interestingly, the male-only dataset is the only place where the smoking status and the diabetes status make up the top 10 features. Age, body mass index, and cholesterol, either HDL or total, are consistently the top three features, regardless of sex. The ECG features of the PQ interval and T-axis appear in all three categories as well.

Table 5 shows the top 10 features based on the SHAP value for the tuned XGBoost model prediction of cardiovascular disease. Across all groups, age is the most important feature in predicting risk. When trained on both sexes, the body mass index and HDL cholesterol also appear for all disease groups. Sex is the most important for ischemic heart disease, but interestingly, it is not in the top 10 for conduction disorders. Out of the traditional risk factors in the Framingham Risk Score, the diabetes status appears only once for the prediction of hypertension and the smoking status does not appear at all. A measure of blood pressure also only appears for any disease and hypertension. The ECG, pulse wave analysis, and magnetic resonance imaging features of the PQ interval, T-axis, pulse rate, R-axis, QRS duration, and LV ejection fraction also appear in two disease categories, each in the top 10.

**TABLE 5:** Top 10 features to predict the risk of cardiovascular diseases for each sex and disease group. Rankings are reported using SHAP on the XGBoost classifiers trained and tuned on each of the 12 datasets with all features from Table 2. BMI, body mass index; BP, blood pressure; HDL, high-density lipoprotein; IM, intima–media; LV, left ventricle; PW, pulse wave.

	Any disease	Hypertension	Ischemic	Conduction
Both sexes	Age	Age	Age	Age
	BMI	BMI	HDL	PQ interval
	Cholesterol	Cholesterol	Sex	QRS duration
	HDL	HDL	Cholesterol	T-axis
	Sex	Central systolic BP	Pulse rate	QTC interval
	T-axis	Sex	T-axis	LV end systolic volume
	QTC interval	R-axis	BMI	P duration
	PQ interval	Mean arterial pressure	LV ejection fraction	HDL
	Systolic brachial BP	Diabetes status	R-axis	BMI
	QRS duration	QTC interval	LV end-systolic volume	Pulse rate
Female only	Age	Age	Age	Age
	BMI	BMI	HDL	HDL
	HDL	Central systolic BP	Max carotid IM thickness 240	QTC interval
	Cholesterol	HDL	Cholesterol	P duration
	QRS duration	Mean arterial pressure	T-axis	T-axis
	T-axis	R-axis	BMI	QRS duration
	Central systolic BP	Cholesterol	Pulse rate	Cent. augment. press
	Mean carotid IM thickness 150	QRS duration	PW arterial stiff. index	PQ interval
	PQ interval	Systolic brachial BP	Mean carotid IM thickness 120	Augmentation index
	PW reflection index	End systolic BP	Mean carotid IM thickness 210	P-axis
Male only	Age	Age	Age	Age
	BMI	BMI	Cholesterol	T-axis
	Cholesterol	Cholesterol	HDL	QTC interval
	QTC interval	Central systolic BP	PW stroke volume	PQ interval
	T-axis	QTC interval	Pulse rate	QRS duration
	HDL	Systolic brachial BP	T-axis	PW stroke volume
	PQ interval	Diabetes status	BMI	BMI
	Smoking status	R-axis	LV stroke volume	Pulse rate
	R-axis	PQ interval	Max carotid IM thickness 210	LV end systolic volume
	Diabetes status	Cent. augment. press	Diastolic BP	P-duration

#### 3.5.1 Female-only dataset

HDL cholesterol is the only other feature to appear in all categories, while the body mass index, total cholesterol, and T-axis appear three times. The smoking and diabetes status do not appear at all. Hypertension is heavily predicted by four different measures of blood pressure, while ischemic heart disease is predicted by several measures of intima–media thickness from carotid ultrasounds. Lastly, the ECG feature, PQ interval, appears in two categories.

#### 3.5.2 Male-only dataset

The body mass index is the only other feature to appear in all categories, while T-axis, total cholesterol, and the QTC interval appear in three of the disease categories. Six of the features for any disease are traditional risk factors. The other four are ECG features. For hypertension, blood pressure measures and diabetes status, along with traditional risk factors, like the age, body mass index, and cholesterol, contribute to risk prediction. Interestingly, three ECG features also make up the top 10 features. For ischemic diseases, the stroke volume and a carotid ultrasound feature add to the traditional risk factors of age, cholesterol, HDL cholesterol, body mass index, and blood pressure. Pulse rate and T-axis conclude the top 10. For conduction disorders, five of the features are ECG features. Age and body mass index are the only traditional risk factors. Magnetic resonance imaging and pulse wave analysis features of the pulse rate, stroke volume, and LV end-systolic volume are the rest of the top 10.

#### 3.5.3 Both sexes combined

Traditional risk factors make up 47.5% of the top 10 features, while magnetic resonance imaging features make up 7.5%, carotid ultrasound 0%, ECG 32.5%, and pulse wave analysis 12.5%. When broken down by sex, for women, traditional risk factors contribute 37.5%, magnetic resonance imaging 0%, carotid ultrasound 10%, ECG 30%, and pulse wave analysis 22.5% to the top 10. For men, the breakdown is traditional risk factors 40%, magnetic resonance imaging 5%, carotid ultrasound 2.5%, ECG 32.5%, and pulse wave analysis 20%.

## 4 Discussion

Women are traditionally underdiagnosed for cardiovascular diseases. The lack of sex-specific criteria is one factor contributing to the underdiagnosis of cardiovascular diseases in women compared to that in men (St. Pierre et al., 2022). From Figure 2, we conclude that in the UK Biobank database, women are nearly 2× more underdiagnosed than men for a first-degree AV block and 1.4× more for dilated cardiomyopathy when using standard sex-neutral criteria. When accounting for average sex differences in the PQ interval, left ventricle diameter, and ejection fractions, the fraction by which women are underdiagnosed would increase even further.

For essential primary hypertension, based on the current sex-neutral criteria, women and men are equally underdiagnosed. Yet, as Figure 2 suggests, women, on average, have lower systolic and diastolic blood pressures than men. If sex-specific criteria were used, women would be underdiagnosed for hypertension. Lastly, women have a smaller wall thickness than men, but the criteria for diagnosing hypertrophic cardiomyopathy are the same. Here, women would again benefit from sex-specific criteria.

The novel SAINT model outperforms XGBoost in predicting the risk for cardiovascular disease. Using a dataset of UK Biobank patients who underwent cardiovascular clinical tests, we designed 60 classifiers based on relevant features, sex, and disease categories. We compared the new deep learning model SAINT to the state-of-the-art approach for tabular data, XGBoost, and to an MLP deep learning baseline. We found that SAINT showed the highest cardiovascular disease prediction AUC in nearly every case, XGBoost typically achieved the second-best AUC, and MLP, the lowest AUC.

SAINT is specifically designed for tabular data, which makes its purpose-driven architecture significantly better for our task than an out-of-the-box MLP. The best performance of SAINT classifiers can be attributed, at least in part, to the fact that our data are composed of numerical, continuous features, which are known to favor the performance of SAINT over classical approaches, such as XGBoost (Borisov et al., 2022). The MLP trained with all cardiovascular and Framingham Risk Score features outperformed the state-of-the-art XGBoost method trained with Framingham-only features for all but two dataset variants, which indicates that having access to more features significantly increases model fidelity for this dataset.

To date, the SAINT architecture has not been applied for risk analysis in cardiovascular diseases. Its remarkable performance not only holds promise for further clinical studies of both cardiovascular diseases and other conditions but also suggests that deep learning approaches could re-surface as viable methods for tabular clinical data modeling. Since deep learning frameworks require large datasets for effective training, we expect SAINT to improve even more as the size of the available medical dataset increases.

Not all traditional risk factors are equally important. Age, body mass index, HDL and total cholesterol, and systolic blood pressure were the most common factors across sex and disease, with the smoking and diabetes status present only for men, as Table 5 suggests. A previous cardiovascular risk prediction study from the UK Biobank used a machine learning pipeline that included 423,604 participants and 473 features, including the Framingham Risk Score, health and medical history, lifestyle and environment, blood assays, physical activity, family history, physical measures, psychosocial factors, dietary and nutritional information, and sociodemographics (Alaa et al., 2019). This group did not have access to cholesterol levels at the time of their analysis, but they did find that the top feature for men and women was age (Alaa et al., 2019), which we reported as well in Table 5. The previous study reported the smoking status and systolic blood pressure in the top 10 for both women and men (Alaa et al., 2019), while we found the smoking status only for men and central systolic blood pressure for women.

When broken down by the disease category, for hypertension measures of blood pressure, the body mass index, age, and cholesterol ranked highly. For men, the diabetes status was an important feature but not for women. Interestingly, only total cholesterol, not HDL cholesterol, ranked in the top 10 for men, while both appeared for women. For ischemic diseases, age, HDL and total cholesterol, and body mass index were in the top 10 for both women and men. For conduction disorders, for women, age and HDL cholesterol were in the top features, while for men, the age and body mass index appeared. The traditional risk factors in the Framingham Risk Score appear to be the most important for a general calculation of cardiovascular risk, but our study suggests re-evaluating it by taking into account the sex- and disease-specific categories. However, the underlying distributions for the traditional risk factors for the male and female populations are significantly different for all factors, except for systolic blood pressure, likely impacting the feature rankings.

ECG recordings are the most effective feature to augment the Framingham Risk Score. For women, traditional risk factors made up 37.5% of the top 10 features, while for men, they made up 40%, as we conclude from Table 5. ECG features appeared next in the top 10, making up 30% and 32.5% for women and men, respectively, followed by pulse wave analysis with 22.5% and 20%, respectively.

ECG features have previously been shown to be powerful predictors of cardiovascular disease (De Bacquer et al., 1998; Raghunath et al., 2021; Khurshid et al., 2022). For instance, the measurement of T-wave morphological variations only requires a single-beat, single-lead ECG and is fast, safe, and shown to identify individuals at risk for sudden cardiac death and life-threatening ventricular arrhythmias (Ramírez et al., 2022). ECG features, such as the QRS duration, QT duration, and T-wave morphology, are associated with increased cardiovascular mortality (Sahli Costabal et al., 2020; Siegersma et al., 2022; Hughes et al., 2023). Women are known to have a shorter PQ interval and QRS duration, longer QTC, and different T-wave morphology than men (Peirlinck et al., 2021a; Siegersma et al., 2022). All of these features appeared in the top 10 from our feature importance analysis across several sex and disease categories, as shown in Table 5. Adding the ECG features with high SHAP values to the traditional Framingham Risk Score features would be a simple yet effective strategy to increase the predictive potential of cardiovascular disease models.

Central blood pressure is more predictive of the cardiovascular disease risk than brachial blood pressure. Pulse wave analysis provides multiple measures of blood pressure. In multiple sex and disease categories, as shown in Table 5, the central systolic blood pressure ranked higher than the systolic brachial blood pressure. Central blood pressure relates closely to the load on the coronary and cerebral arteries and, as such, is more strongly correlated with vascular diseases and negative outcomes than brachial blood pressure (Roman et al., 2007). Other pulse wave features, like the pulse rate, arterial stiffness index, reflection index, and mean arterial pressure, that made up the top 10, as shown in Table 5, have also previously been linked to an increased risk of cardiovascular disease (Kengne et al., 2009; Mitchell, 2009; Benetos et al., 2012; Cecelja and Chowienczyk, 2012).

Carotid ultrasounds provide an accessible way to monitor ischemic heart diseases. Carotid ultrasounds measure the carotid intima–media thickness; a thicker intima–media thickness may indicate atherosclerosis of the carotid artery, leading to the brain (Bots et al., 1997). Increasing evidence suggests that atherosclerosis in the carotid artery is associated with atherosclerosis in the coronary artery, leading to an increased risk of stroke, myocardial infarction, and other ischemic heart diseases (Bots et al., 1997; Zhao et al., 2016; Bytyçi et al., 2021). Because the carotid artery is easily accessible compared to the coronary artery, carotid ultrasounds provide a non-invasive, simple way to screen patients for the increased risk of cardiovascular diseases (Bytyçi et al., 2021). In Table 5, we found that three features from the carotid ultrasound for women and one for men appeared in the prediction of ischemic diseases. One carotid ultrasound feature also appeared for women for the prediction of any cardiovascular disease. As such, carotid ultrasounds provide valuable insights on an individual’s risk for ischemic diseases, regardless of the sex, and may be especially useful for monitoring the cardiovascular disease risk of women in general.

Limitations and future work: Our study provides a first step toward rethinking about risk indicators for cardiovascular diseases in view of big data and machine learning. Although our results provide encouraging evidence of the added value of leveraging both technologies, it is important to be aware of the limitations to our current approach and, ideally, address them in future follow-up studies. First, the Framingham score was designed to provide a 10-year prediction of risk for developing cardiovascular disease (Lloyd-Jones et al., 2004). Instead, here, we have used these features for the *detection* of cardiovascular diseases, to identify when it is currently present in an individual. A follow-up study with later time points would be needed to determine which features are best for a 10-year *prediction* of cardiovascular disease. Second, although the population of individuals with ICD-9 data is only 4% of the entire UK Biobank population, by not including these additional medical data along with the ICD-10 data that are used in this study, there may be valuable information missing on the prevalence of cardiovascular diseases in certain populations. Third, and most notably, the outcome of our approach is only as good as the clinical diagnoses that define our classification. It would be highly beneficial, and actually very feasible with modern machine learning techniques, to perform a comprehensive study of the human-level error by evaluating expert clinician performance on the utilized datasets. This would provide an estimate of the Bayes error rates for our 12 datasets that could then be compared to the SAINT model performance. Fourth, the population of the UK Biobank is fairly homogeneous, so our classifiers might not be generalizable to participants outside the United Kingdom who are from more diverse racial and ethnic backgrounds. Fifth, since SAINT was the best-performing approach for our classification tasks, future studies could focus on integrating a comprehensive feature importance pipeline, such as SHAP, into the SAINT model evaluation. This would leverage the high performance of the SAINT method and could translate to even more informative and credible feature significance rankings. Finally, a direct comparison between the XGBoost and SAINT feature analyses would provide further insights into the sensitivity of feature identification with respect to the specifics of a given learning architecture.

## 5 Conclusion

Women are underdiagnosed for cardiovascular diseases compared to men. Unarguably, there is an urgent need for sex-specific diagnostic criteria. Deep learning provides powerful tools to precisely quantify how well traditional risk factors, like the Framingham Risk Score, predict the risk of cardiovascular diseases, for females, males, or both sexes combined. Alarmingly, our deep learning study revealed that, for a first-degree atrioventricular block and dilated cardiomyopathy, women are underdiagnosed 2× and 1.4× more than men. Inversely, without much extra work, our deep learning approach allows us to identify and rank the most predictive features for different types of cardiovascular diseases, sex specifically and sex neutrally. We found that, out of the four commonly used clinical tests—electrocardiograms, magnetic resonance imaging, carotid ultrasounds, and pulse wave analysis—electrocardiogram features showed the most promise in increasing cardiovascular disease prediction. A more accurate individualized risk prediction of cardiovascular diseases would enable personalized treatment and prevention strategies, a more effective allocation of medical resources, and an early and precise identification of high-risk individuals, toward the ultimate goal to improve patient outcomes, reduce morbidity and mortality, and improve the quality of life.

## Data Availability

The datasets presented in this article are not readily available because researchers must apply to access the UK Biobank dataset. Requests to access the datasets should be directed to https://www.ukbiobank.ac.uk/.

## Appendix A Hyperparameter tuning

MLP: A deep learning model for baseline comparison. We apply Bayesian optimization using the Keras Tuner to tune the hyperparameters of the multilayer perceptron over the validation data for the dataset with both sexes and any disease. The optimization shows that a multilayer perceptron architecture with six hidden layers, 30 units in each hidden layer, and an *L*_2_-regularization parameter *λ* = 10^–2^ in each layer provides a robust model performance. We observe that a batch size of 32 yields the fastest convergence since all datasets are small-to-medium-sized. A maximum of 100 epochs allows us to reach convergence while preserving the computational feasibility. We further conclude that the performance of the multilayer perceptron on the validation data is not highly sensitive to changes in other hyperparameters, so we used the default values of the learning rate and the coefficients in the Adam update rule. In principle, we should apply this hyperparameter study to each of the 12 multilayer perceptrons individually. However, the Bayesian tuning process itself is parallelized on a single GPU, and we decided not to tune all 12 multilayer perceptron models individually. We applied the hyperparameters of both sexes, any disease dataset, to the remaining 11 models.

XGBoost: A state-of-the-art analysis of tabular data. Since XGBoost training is significantly faster than multilayer perceptron training and is readily parallelizable over the 12 dataset variants, we apply a random search with five-fold cross-validation to tune the hyperparameters of each of the XGBoost models individually. Specifically, we tune the XGBoost hyperparameters for each of the 12 dataset variants, and for the two input feature groups, all available features (cardiovascular and the Framingham Risk Score), and Framingham Risk Score features only. Based on prior work (Putatunda and Rama, 2018; Gupta et al., 2020), the most valuable hyperparameters for tuning include the maximum tree depth, learning rate, subsample ratio of the training instances, subsample ratio of columns for each tree, subsampling ratio of columns for each level, and the number of tree estimators. We tune these hyperparameters in a parallelized random search routine. The tuning process minimizes the AUC score over the validation data. We compare two different XGBoost ensembles: one set that has access to all features (cardiovascular and Framingham Risk Score features) and another set that can train on Framingham Risk Score features and the body mass index only. Each set of XGBoost models is applied to all 12 dataset variants, and hyperparameter tuning is performed for each XGBoost model individually. Thus, we compute 24 optimal sets of XGBoost hyperparameters for the resulting 24 population–disease–feature combinations (three input population groups, four disease label sets, and two feature groups). For evaluation purposes, we consider both the 24 tuned and 12 untuned XGBoost models separately, which yield a total of 36 XGBoost ensembles for performance analysis. The 12 untuned XGBoost models for the Framingham Risk Score-only feature group are not included in the evaluation due to redundancy.

SAINT: A novel approach for tubular learning. We did not have access to sufficient computed data to be able to perform full-scale hyperparameter tuning for the 12 SAINT classifiers. We did, however, make adjustments to hyperparameters that proved to have the most significant effect on the validation performance. Specifically, we increased the weight decay parameter of the AdamW optimization scheme (Loshchilov and Hutter, 2019) to *w* = 10 since lower values (such as the default of *w* = 10^–2^) led to significant overfitting on the training set. We also found that the default choices of the learning rate and other AdamW optimization parameters provided the best validation set performance.

## Appendix B Computational structure of the SAINT architecture

Here, we present a brief synthesis of the SAINT framework structure, as described by Somepalli et al. (2021). From a high-dimensional embedding of the input features, a single stage of the SAINT unit computes the output through a series of multi-head self-attention blocks, intersample attention blocks, feed forward layers, and layer-normalization layers. Several of these stages are stacked sequentially before the final contextual representation is generated. In each stage, the self-attention block applies attention among the features of a given sample, while the intersample attention applies row-wise attention across different samples for a given feature. Tabular data learning in SAINT is divided into two phases: self-supervised pre-training and supervised fine tuning. The pre-training phase consists of minimizing the combined contrastive features and denoising the cost function without considering the example labels. The fine-tuning phase evaluates a deviation metric between the ground truth and model prediction. Throughout this study, we perform a binary classification of cardiovascular disease presence and use the binary cross-entropy loss function as our deviation metric of choice.
